# Objective assessment of surgical operative performance by observational clinical human reliability analysis (OCHRA): a systematic review

**DOI:** 10.1007/s00464-019-07365-x

**Published:** 2020-01-17

**Authors:** Benjie Tang, Alfred Cuschieri

**Affiliations:** 1 Surgical Skills Centre, Ninewells Hospital and Medical School, Level 5, School of Medicine, University of Dundee, Dundee, DD1 9SY Scotland, UK; 2 Institute for Medical Science and Technology, School of Medicine, University of Dundee, Dundee, Scotland, UK

**Keywords:** Observational clinical human reliability analysis (OCHRA), Objective assessment of surgical operative performance, Technical error, Task performance, Hazard zones of operations, Proficiency–gain curves

## Abstract

**Background:**

Both morbidity and mortality data (MMD) and learning curves (LCs) do not provide information on the nature of intraoperative errors and their mechanisms when these adversely impact on patient outcome. OCHRA was developed specifically to address the unmet surgical need for an objective assessment technique of the quality of technical execution of operations at individual operator level. The aim of this systematic review was to review of OCHRA as a method of objective assessment of surgical operative performance.

**Methods:**

Systematic review based on searching 4 databases for articles published from January 1998 to January 2019. The review complies with Preferred Reporting Items for Systematic reviews and Meta-Analyses (PRISMA) guidelines and includes original publications on surgical task performance based on technical errors during operations across several surgical specialties.

**Results:**

Only 26 published studies met the search criteria, indicating that the uptake of OCHRA during the study period has been low. In 31% of reported studies, the operations were performed by fully qualified consultant/attending surgeons and by surgical trainees in 69% in approved training programs. OCHRA identified 7869 consequential errors (CE) during the conduct of 719 clinical operations (mean = 11 CEs). It also identified ‘hazard zones’ of operations and proficiency–gain curves (P-GCs) that confirm attainment of persistent competent execution of specific operations by individual trainee surgeons. P-GCs are both surgeon and operation specific.

**Conclusions:**

Increased OCHRA use has the potential to improve patient outcome after surgery, but this is a contingent progress towards automatic assessment of unedited videos of operations. The low uptake of OCHRA is attributed to its labor-intensive nature involving human factors (cognitive engineering) expertise. Aside from faster and more objective peer-based assessment, this development should accelerate increased clinical uptake and use of the technique in both routine surgical practice and surgical training.

Traditionally, the quality of surgery is assessed on morbidity and mortality data (MMD) [1, 2]. Useful as it is in hospital surgical practice, the limitation of MMD as a performance index, is its retrospective nature. *Learning curves* (LC) are often used by surgeons who are ‘learning’ (i.e., gaining proficiency) in the execution of an operation, as performance improves with increasing experience [3-6].

Neither MMD nor LCs can provide objective information on the nature of intraoperative errors and their mechanisms when these effect adversely patient outcome. Specifically, they fail to differentiate the exact role of technical errors from other components of surgical competence, e.g., non-technical skills [7-10], or the level of proficiency of surgeons by proficiency–gain curves (P-GCs) (Fig. 1). The P-GC of an individual surgeon for an operation represents the time course on repeat executions through which the trainee reaches the proficiency zone and is then able to perform the operation consistently well with good patient outcome; benchmarked by Surgical Colleges and required by Credentialing Committees and National Licensing Bodies. In essence, these safeguard society from surgeons who cannot or have lost the ability to operate safely and to the ‘accepted standard of care’ [7]. The underlying root causes of the adverse events are technical errors which often also provide key information on learning opportunities to prevent or reduce adverse events [11-14].

**Fig. 1:**
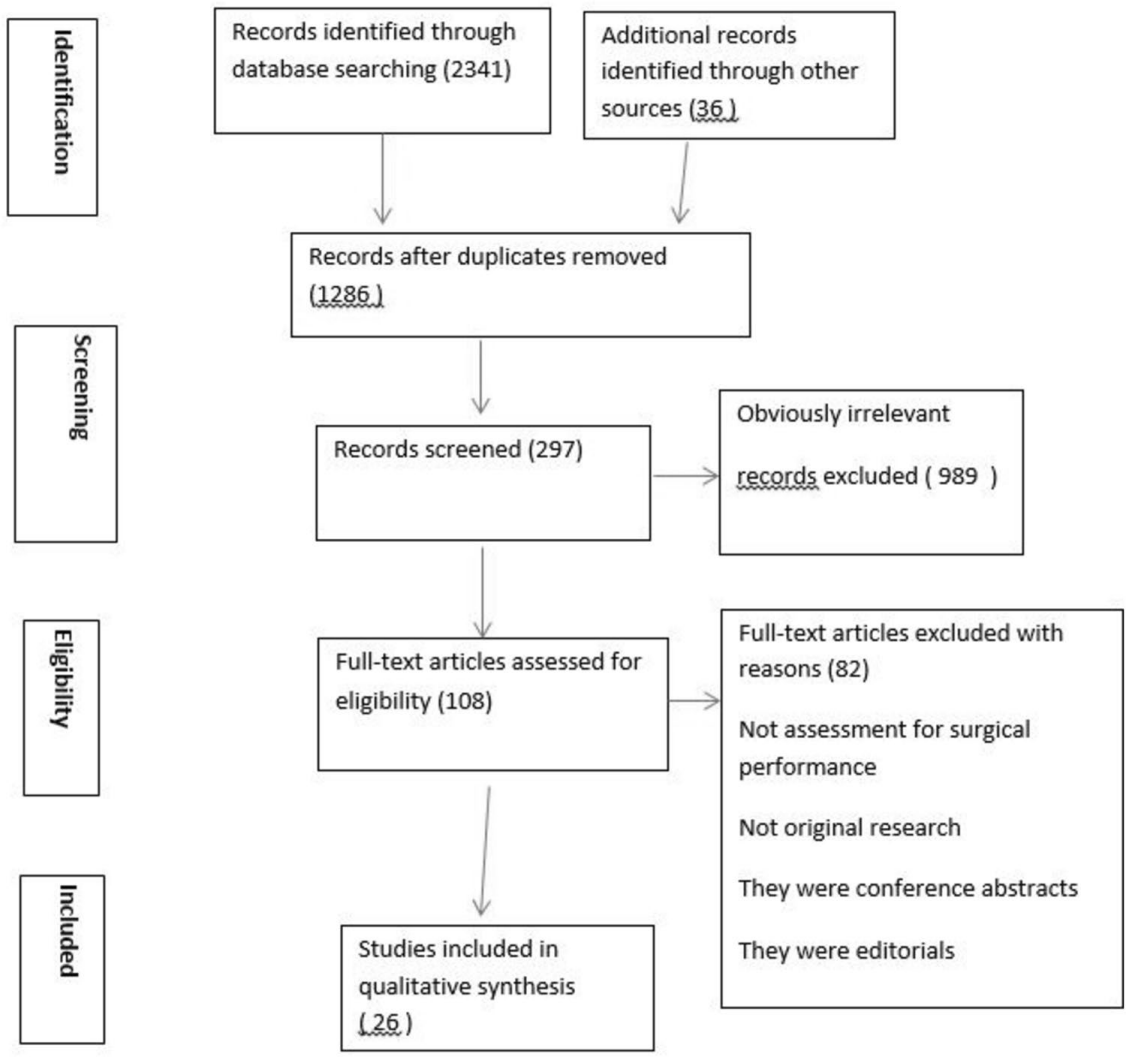
PRISMA guidelines-based selection of publications for systemic reviews

An alternative approach to human error reduction is human reliability analysis (HRA) techniques [15-20]. These are widely used in risk management of safety–critical systems, e.g., nuclear power industry, aviation industry, and military operations. HRA techniques determine the impact of human error within a system. The techniques are those of systems engineering and cognitive and behavioral science. They are used to analyze and understand the human contribution to the system’s reliability and safety [19, 20]. Common steps of the HRA process consist of problem definition and specification of the task and its modeling, human error identification and analysis, human error quantification, and error management.

The first study to use of HRA techniques in laparoscopic surgery was published in 1998. It analyzed the surgical task performance based on technical errors during laparoscopic cholecystectomy (L chole) [15]. Subsequent research from the Surgical Skills Unit in Dundee was directed towards increasing the clinical relevance of HRA. This was necessary as HRA is essentially predictive, i.e., its objective being to ensure that the activity, e.g., civilian flight, space flight etc., is as safe as is humanly possible before the aircraft takes off. In sharp contrast, all operations can nowadays be assessed objectively from an unedited video recording using established human factors (cognitive) engineering expertise. This approach renders HRA observational and specific to an operator. Hence this modified HRA is referred to as ‘Observational Clinical – Human Reliability Assessment (OCHRA) [16, 21-42]. The purpose of this review was to analyze the current state, uptake and limitations of the use of OCHRA to assess intraoperative technical errors, hazard zone of operations and proficiency–curves of operations.

## Methods

### Search strategy and criteria

The review was performed using the guidelines outlined in Systematic Reviews and Meta-Analyses (PRISMA) statement (Fig. 1) [43]. Only publications related to assessment of surgical task performance and surgical operations by identifying technical errors using HRA and OCHRA were included across specialties: General Surgery, Colorectal Surgery, Bariatric Surgery, Urology, Ophthalmic Surgery, Pediatric Surgery, and Otorhinolaryngology. Surgical tasks in surgical training programs and surgical performance in experimental surgical studies were also included. Exclusions were publications on non-surgical performance, descriptive publications without data, conference abstracts, letters, editorials and commentaries, and non-English publications.

Since this study was a systematic review and there were no human subjects involved, thus, the institutional review board (IRB) approval and written consent were not required.

### Eligibility criteria

An initial search was carried out on PubMed, EMBASE, Web of Science and the Cochrane Library for English language articles published from January 1998 to January 2019. Search strategy and terms used included ‘human reliability analysis (HRA),’ ‘observational clinical human reliability analysis (OCHRA),’ ‘human error in surgery,’ ‘adverse events,’ ‘human error identification,’ ‘technical error in surgery,’ ‘surgical performance,’ ‘task analysis in surgery,’ and ‘competency assessment.’ A further search used terms such ‘patient safety,’ ‘hazard zones in surgery,’ ‘human factors in surgery,’ ‘proficiency–gain curves in surgery,’ ‘surgical skills training.’ All the key search terms were combined subsequently.

The culled publications were retrieved in full text for further assessment for eligibility. Following review, relevant references cited in the included articles were also retrieved and scrutinized.

### Data extraction and synthesis

Studies describing use of HRA or OCHRA for direct assessment of surgical operations were grouped together. Other publications in which HRA or OCHRA were used as one of the methods to assess surgical task performance for research projects were grouped separately. Microsoft Excel 2016 (Microsoft Corp, Redmond, WA) was used to manage the extracted data. Risk of bias within individual or across studies was not specifically assessed.

### Assessment of methodological quality

The Medical Education Research Study Quality Instrument (MERSQI) [44] was applied to assess the quality of studies conducted using OCHRA. The MERSQI contains 10 items that reflect 6 domains of study quality including study design, sampling, type of data, validity, data analysis, and outcomes. MERSQI produces a maximum score of 18 with a potential range from 5 to 18. The maximum score for each domain is 3. The overall MERSQI scores pf the publications included in the review are shown in Table 1.

**Table 1:** Synthesis and analysis of publications on HRA and OCHRA

Year	Author	Study setting/model used	Aim of study	Assessment	Influencing factor/underling mechanism	Outcome/comment	MERSQI
Use of HRA or OCHRA for direct assessment of surgical procedures							
1998	Joice et al.	Clinical setting (CS): Laparoscopic cholecystectomy (LC)	Task analysis and technical errors documentation	HRA: first application of HRA to analyze surgical performance in the literature	External Error Modes (EEM)	189 technical errors identified in 20 laparoscopic cholecystectomy. error frequency with tasks and in error frequency in the use of different instruments	12
2003	Malik et al.	Clinical setting: Endoscopic dacryocystorhinostomy	Detection of technical errors and external error mode (EEM)	HRA	EEM	Specific technical error resulted in nasal mucosa noted in 12 operations. execution error such as “too much force “was most commonly enacted	10
2004	Tang et al.	Clinical setting: Laparoscopic cholecystectomy	Identification and categorization of consequential and inconsequential technical errors, error probability, hazard zone, and EEM	OCHRA: first introduced and published OCHRA in the surgical literature	EEM	38,062 movements within 200 LC performed by 26 surgeons analyzed and 2242 technical errors identified. consequential and inconsequential error, error probability within tasks, and concept of hazardous zone were first defined and categorized	15
2004	Tang et al.	Clinical setting: Laparoscopic pyloromyotomy (LPM)	Technical error analysis, error probability, and hazard zone	OCHRA	EEM	50 cases of LPM (310 errors in 50 videotapes) analyzed—number of errors committed with different instruments, EEM underlying errors committed with different instrument, error probability for different tasks, hazard zones of an operation—50 videotapes	13
2005	Tang et al.	Laboratory setting: LC	Procedural and execution error, error pattern and error probability with specific instruments	OCHRA: Surgical performance of 60 surgical trainees analyzed	EEM: The important underling factors for the trainee errors were (1) omission or wrong sequence important steps (72%), (2) use of excessive or too little force (38%)	60 lab-based laparoscopic cholecystectomy analyzed and 1067 technical error identified including 331 consequential errors. The underling factors for errors were: (1) omission of important steps, (2) execution of steps in the wrong sequence, and (3) use of excessive force. These 3 types of errors accounted for 92% of consequential errors and 57% of inconsequential errors. The error probability committed by trainees was 2 times higher than experts	10
2008	Cox et al.	Clinical setting: cataract surgery	Identify inconsequential error and document the most common error enacted during the phacoemulsification	HRA	EEM: Step is not done and step is done with too much force	Sixteen phacoemulsifications analyzed. 84 total error with 7 consequential errors in 16 consecutive phacoemulsification. The commonest single error was difficulty in “cracking” the nucleus	10
2008	Gauba et al.	Clinical setting: cataract Surgery	Technical errors analysis	HRA	EEM	Analysis of 330 constituent steps of 33 operation identified 228 errors, of which 151(66.2%) were executional and 77 (33.8%) were procedural. The finding of high executional error rate could be used to enhance and structure resident surgical training and future assessment tool	9.5
2009	Talebpour et al.	Clinical setting: Laparoscopic anastomosis	To study a proficiency curve of advanced laparoscopic anastomosis using OCHRA	OCHRA: First proficiency curve identified in the surgical literature	EEM: the important performance-shaping factors identified were: concentration lapse (n-1,321), misjudgments (n = 209), poor camera work (n = 193), fatigue (n = 1280, and impaired coordination (n = 108)	Twenty laparoscopic anastomosis performed and analyzed. For this surgeon proficiency in execution laparoscopic palliative bypass was reached after the 14th anastomosis when efficient execution was accompanied by significant reduction in technical errors and improved economy of movement	12
2012	Ahmed et al.	Clinical setting: Laparoscopic Roux-en-Y gastric bypass	Root cause analysis of internal hernia and Roux Limb compression	OCHRA	EEM: Missing intermesenteric stitches on both side of the Roux limb	Forty-six laparoscopic Roux-en-Y were analyzed. More errors occurred in the complication groups than in the control group(Internal hernia 5.85, Roux compression 3.53, control 0.90, *P* < 0.001)	11.5
2012	Miskovic et al.	Clinical setting: Competency assessment in laparoscopic colorectal surgery at the specialist level	Evaluated construct and concurrent validity of OCHRA for competency assessment at a specialist level	OCHRA: First time of use of OCHRA for competency assessment at a specialist level. Thirty-two consultant surgeons were assessed	EEM: Execution error accounted 73% of consequential errors	399 technical errors identified in 32 cases performed by consultant surgeons. Delegates committed higher error rate than experts in tissue handling. Delegates had significantly dissection /exposing time ratio. OCHRA is a valid tool for assessing competency at a specialist level in advanced surgery	15
2014	Mendez et al.	Clinical setting: Neck dissection	Use of OCHRA to validate on high definition video teaching module for learning neck dissection	OCHRA	EEM	Six Residents each performed 2 operation. Residents performed significantly less errors following exposure to the high definition video module	8.5
2016	Foster et al.	Clinical setting: laparoscopic rectal cancer surgery	To investigate the application of OCHRA technique for assessing technical performance of laparoscopic rectal surgery and explore the validity and reliability of OCHRA	OCHRA: First study of use of OCHRA to analyze one of the most complex procedure in surgery	EEM: dominantly execution errors identified	335 execution errors identified in 20 cases. More error were observed during the pelvic tasks compared with abdominal tasks (*P* < 0.001). Within the pelvis, more errors were observed during dissection on the right ride than the left (*P* < 0.03) OCHRA offers a valid and reliable method for evaluating technical performance of laparoscopic rectal surgery	14
2017	Rutte et al.	Clinical setting: Laparoscopic sleeve gastrectomy	To detect the key elements of the sleeve gastrectomy and find the potential hazard zones of the procedure	OCHRA	EEM	A total of 213 technical identified in 60 sleeve gastrectomy (SG) procedure. 44.6% were consequential error in which 96 errors required additional actions. 13 key steps of the SG were defined. Most consequential errors enacted during the dissection of the greater curvature and during stapling of the stomach	12.5
2017	Hamour et al.	Clinical setting: thyroidectomy surgery	Use of OCHRA to validate a high definition video-based teaching module instructing thyroidectomy surgery to Otolaryngology-Head and Neck surgery trainees	OCHRA	EEM	Six participant performed 6 cases of thyroidectomy. The mean technical error rate was 8.8 errors per procedure before module exposure and 4.5 error per procedure after exposure	8.5
Use of HRA or OCHRA technique as one of the techniques for assessment of task performance for other surgical researches							
2004	Alijani et al.	Clinical setting: Laparoscopic cholecystectomy	To compare intraoperative cardiac function, postoperative cognitive recovery, and surgical performance of LC with abdominal wall lift (AWL)versus positive pressure capnoperitoneum (PPCpn)	HRA, Doppler machine, an auditory vigilance test. First time to use HRA for surgical scientific research in the literature	None	40 operations was randomized into AWL and PPCpn. The AWL group had significantly higher instrument movements (550 ± 57 versus 198 ± 21; *P* = 0.00001), and higher number of errors with consequence (7.1 ± 1.1 versus 2.9 ± 0.4; *P* = 0.0001). AWL increases the level of difficulty in the execution of the operation	11.5
2006	Tang et al.	Lab setting: laparoscopic skills course one a wide range of operative skills	To develop a new approach to combine objective structured Clinical Examination (OSCE) and OCHRA to assess operative and cognitive skills during laparoscopic course	OCHRA and OSCE	EEM	Sixty participant recruited. OCHRA provides a discriminative feedback assessment of laparoscopic operative skills. OCHRA and OSCE are best regarded as complementary assessment tools for operative and cognitive skills	11
2008	Mishra A et al.	Clinical setting: team work, technical performance in laparoscopic cholecystectomy	To investigate the relationship between non-technical teamwork skills and technical errors	OCHRA and Oxford NOTECHES	None	Twenty-six LC performed by a team. Non-technical skills are an important component of surgical skill, particularly in relation to the development and maintenance of a surgeon’s situational awareness	9.5
2008	McCulloch et al.	Clinical setting: LC and carotid endarterectomy (CEA)	To study the effects of “non-technical skills” on attitudes, teamwork, technical performance and clinical outcome in LC and carotid endarterectomy (CEA) operations	OCHRA, Oxford NOTECHES, Crew Resource Management (CRM)	None	Twenty-six LC and 22 carotid endarterectomies were studied. Non-technical skills training improved technical performance in theater, but the effects varied between teams. Considerable cultural resistance to adoption was encountered, particularly among medical staff	10
2008	Catchpole et al.	Clinical setting: performance in operating room	To analyze the effects of surgical, anesthetic, and nursing teamwork skills on technical outcome	OCHRA and Oxford NOTECHES	None	Twenty-six LC and 22 carotid endarterectomies were studied. Detailed analysis of team interactions and dimensions is feasible and valuable, yielding important insights into relationships between non-technical skills, technical performance, and operative duration	10
2009	Manasnayakorn et al.	Lab setting: laparoscopic suturing to close a enterotomy	To investigate the influence of the working surface height on task performance and muscle workload in hand-assisted laparoscopic surgery	OCHRA and leakage pressure, visual analogue score, electromyography, and placement error score	None	Ten subjects participated the study. The optimum table height for hand-assisted laparoscopic surgery allows the working surface of the extracorporeal instrument handle to be at or 5 cm above the elbow level	8.5
2015	Ghazanfar et al.	Lab setting: laparoscopic operative tasks	To compare the effect of dividing attention of novices and experts on a laparoscopic task performance	OCHRA and operating time	None	Thirty-four subjects participated the study. 21,109 movement and 9036 errors were analyzed. Novices had increased mean task completing time (second) (171 ± 44 vs 149 ± 34, *P* < 0.05), number of errors (127 ± 51 vs. 96 ± 28). Junior surgeons are less able to focus their attention in a divided attention conditions in theater environment	8
2017	Tang et al.	Lab setting: transurethral resection of the prostate (TURP)	To develop and validate a new and cost effective animal tissue model for practicing of TURP	OCHRA, 5-point Likert Scale questionnaire, and operating time	None	Thirty-five subjects were recruited in the study. In the construct validity study, the use of OCHRA identified that trainees committed more technical errors than the expert ( 11 vs 7, *P* < 0.001), produced more instrument movements ( 51 vs 33, *P* < 0.001), and required longer operating time ( 11.4 vs 6.2 min, *P* < 0.001)	11.5
2018	Boghdady et al.	Lab setting: laparoscopic intracorporeal knotting	To study the effect of a self-administered checklist on the laparoscopic task performance of novices during a standardized task	OCHRA used for error analysis	None	Twenty novices were recruited to the study. 2341 were detected during the 5 separate stages. The checklist group committed significant fewer errors as compared with the control group during all the later 4 stages	9.5
2018	Boghdady et al.	Lab setting: tests of spatial coordinates in two-dimensional (2D)and three dimensional(3D) image	To compare generic components of 2D versus 3D laparoscopic images	OCHRA	None	Twenty-four novice subjects participated the experiment. 3D performed more accurately in comparing volumes (*P* < 0.05). In spatial coordinates, there were statistically significant higher number of errors in 2D as compared to 3D (*P* < 0.002)	9.5
2018	Boghdady et al.	Clinical setting: laparoscopic cholecystectomy	To study the effect of a performance-based self-administered intra-procedural checklist on the performance of trainees during elective laparoscopic cholecystectomy	OCHRA and 5-point Likert scale questionnaire	None	Twenty-four novice participants performed statistically better with the application of the checklist compared to wen no checklist was used, respectively: Median (IQR) total number of errors 1.51 (0.80) versus 3.84(1.42) (*P* = 0.002) and consequential errors 0.20(0.12) versus 0.45(0.42) (*P* = 0.005)	11.5
2018	Francis et al.	Clinical setting: laparoscopic surgery	To develop a structure, practical method to report intraoperative adverse events during minimal access surgery procedures	A structured mixed methodology approach, expert opinion, OCHRA	None	Thirty-four European Association for Endoscopic Surgery experts. 217 h of TME surgery were analyzed to develop and continually refine the five-point hierarchical structure. 34 European Association for Endoscopic Surgery experts responded. The observed distribution of intraoperative adverse events was 60.1% grade I (non-consequential), 37.1% grade II (minor Corrective action), 2.4% grade III (Major correction or change in postoperative care) and 0.1% grade IV (life threatening)	8

## Results

**Fig. 2:**
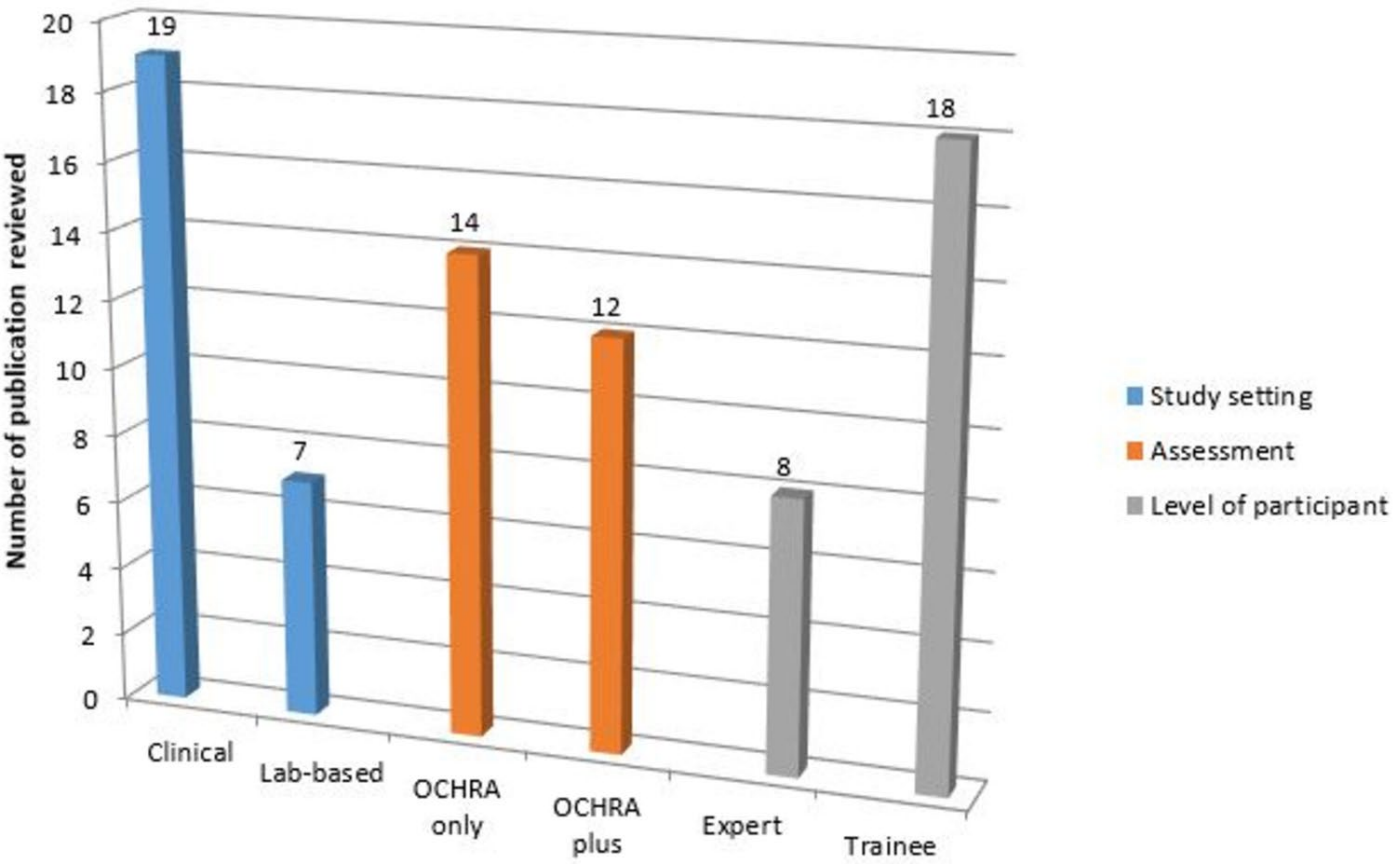
Analysis of published studies included in review

A total of 2341 publications were screened, of which 297 were read in full text. Of these, 82 studies were excluded as not relevant. After the eligibility criteria of inclusion and exclusion were applied, a total of 26 studies were selected in the final data set for analysis (Fig. 1), with the majority (73%) being clinical. Thirty-one percent of these were performed by consultant surgeons and 69% by surgical trainees in established surgical training programs. OCHRA as the only assessment method was used in 54% of the 26 publications (Fig. 2).

OCHRA was applied to 719 surgical operations for direct analysis of the technical errors, hazard zones, external errors modes and P-GCs. The data also included a range of experimental research projects carried out by 265 surgical trainees, the vast majority of which used OCHRA with HRA being used only in 3 publications to evaluate surgical task performance.

Sixteen different surgical operations were analyzed using OCHRA: General Surgery, Colorectal Surgery, Bariatric Surgery, Urology, Ophthalmic Surgery, Pediatric Surgery, and Otorhinolaryngology. During execution of these operations, 7869 consequential errors were identified and analyzed (Table 1). Error rates and external error modes varied depending on the type of operations and level of experience of operators. In general, surgical trainees committed twice as many technical errors as specialist consultant/attending surgeons [16, 22].

The consequential error rate averaged 11 per procedure with a wide range of 4–34 (Table 1) depending on the complexity of the operation and level of expertise and skill of the operator. In one case series of 200 LCs [16], the inter-rater consistency of OCHRA was 85% and a strong correlation was observed between proficiency and error frequency upon test-re-test analysis (*r* = 0.79, *P* < 0.001) [25]. In a similar study evaluating performance in advanced laparoscopic surgery, analysis of 335 execution errors showed a significant correlation between error frequency and mesorectal specimen quality (*R*s = 0.52, *P* = 0.02) and with blood loss (*R*s = 0.609, *P* = 0.004) [25]. Classification of intraoperative adverse events using OCHRA was agreed by 84% of 34 European Association for Endoscopic Surgery (EAES) experts in laparoscopic surgery [19]. Error rates and external error modes varied, depending on the type of operations and level of experience of operators. In general, surgical trainees committed twice the technical error rate than specialists [14, 22].

Only two publications reported on External Error Mode (EEM), both on laparoscopic colorectal resections. The first study reported on EEM at different levels of expertise and was based on 32 video-recorded laparoscopic colorectal resections, performed by experts and delegates of the National Training Program in England [28]. All included errors on tissue-handling, instrument-misuse, and times spent on dissecting (D) and exposure (E). This new performance variable was referred to as the D/E ratio. Two independent expert surgeons globally assessed each video in terms of competency (pass vs. fail). The study identified 399 errors and reported significant differences between expert, pass, and fail candidates for total errors; with median errors for experts, pass, and fail candidates being 4, 10, and 17 (*P* < 0.001), respectively. Comparison between the pass and fail candidates showed more tissue-handling errors in the failed group (7 vs. 12; *P* = 0.005), but not for consequential and instrument-handling errors. As expected, the D/E ratio was significantly lower for delegates than for experts (0.6 vs. 1.0; *P* = 0.001) [28]. In this study all 4 independent variables were used to predict delegates who passed or failed the assessment, the area under the receiver operating characteristic curve was 0.867, sensitivity 71.4%, specificity 90.9%, and overall predictive accuracy 84.4%. Thus, OCHRA provides significant discriminative power (construct validity) between competent and non-competent performance [28].

The second, a single-center study, used OCHRA to identify technical errors enacted in unedited videos of 20 consecutive laparoscopic rectal cancer resections [25]. The study identified 335 execution errors with a median of 15/operation. More errors were enacted during pelvic compared with abdominal steps (*P* < 0.001). Additionally, more errors were observed during dissection on the right than the left side of the pelvis (*P* = 0.03).

Hazard zones and difficult tasks were identified in all major commonly performed laparoscopic operations such as general surgical, colorectal, bariatric and ENT operations [16, 21, 25, 27, 29, 30, 32, 33]. Examples include dissection of triangle of Calot during LChole, dissection of right side of pelvis during laparoscopic resection of rectal cancer, mobilization of the greater curvature and stapling of the stomach during sleeve gastrectomy and access to nasal cavity during endoscopic dacryocystorhinostomy (DCR). Difficult tasks were also identified by OCHRA, e.g., intracorporeal sutured laparoscopic anastomosis and laparoscopic gastric bypass [33, 34].

**Fig. 3:**
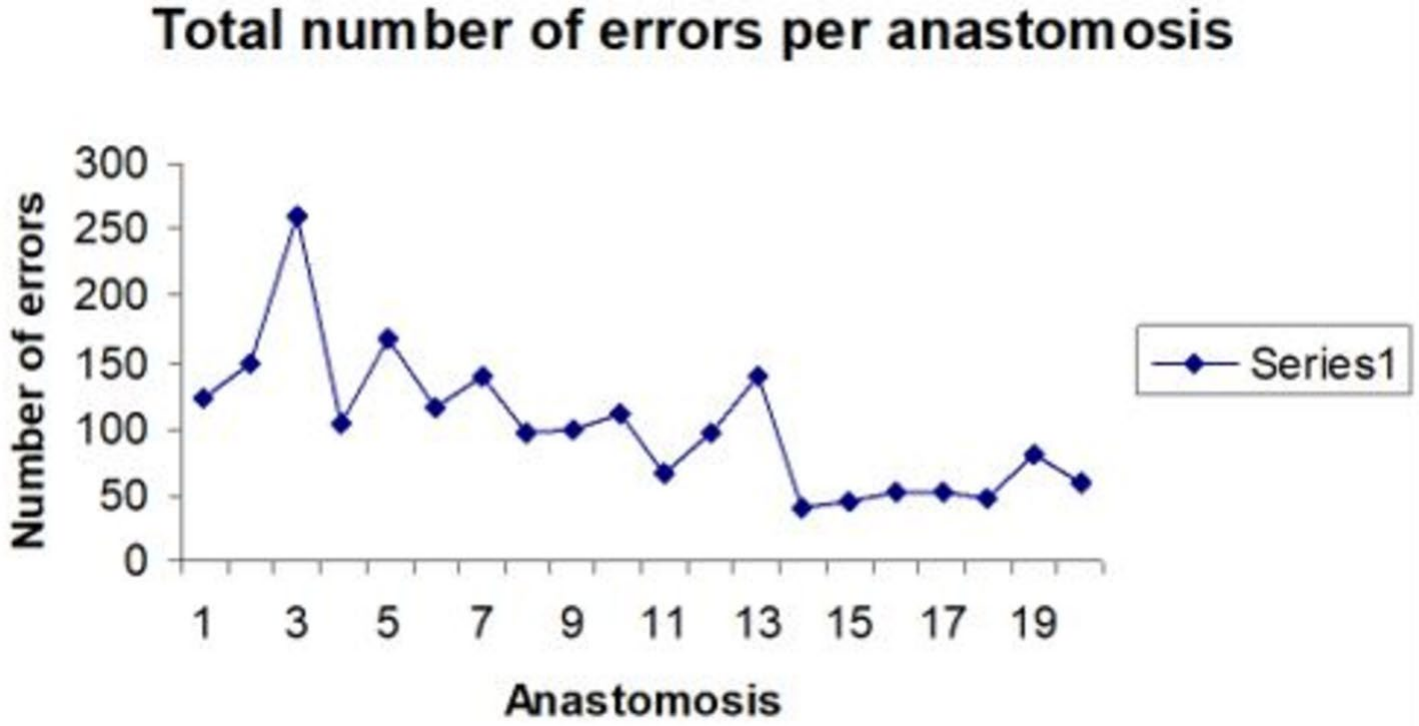
Attainment of proficient execution of palliative laparoscopic bilio-enteric bypass by surgical fellow (MT) indicating that this surgeon needed to perform 13 such procedures to reach a nadir of a few inconsequential operations [34]. Reproduced by permission of Editor in Chief/Publisher of Surgical Endoscopy

The data also confirmed that OCHRA can be used to quantify the P-GC for a laparoscopic operation indicated by reaching the proficiency zone, when the individual surgeon attains maximal optimal performance in the execution of a specific procedure (Fig. 3) [34, 45]. It has also been suggested that OCHRA is a valid tool for assessing competency level in advanced specialist surgery, e.g., laparoscopic colorectal surgery [23, 25, 28].

## Discussion

OCHRA assesses the quality of execution by a surgeon (performance level) by detection and characterisation of technical errors (procedural/execution) and (consequential/inconsequential) enacted by the operator during the operation [16, 21-34]. In this process OCHRA, divides the continuum of an operation into steps, tasks and hazard zones, the last referring to sections of an operation where major errors, some catastrophic, iatrogenic injuries, occur most commonly [16, 21, 25-33].

The reported significant correlation between OCHRA error rates and quality of total mesorectal excision also confirms the clinical relevance of the technique in quality assessment of surgical performance [25, 28]. It also detects the attainment of complete proficiency reached by a surgeon indicated by a nadir of only a few inconsequential errors. This ability of OCHRA is currently underutilized in both surgical training and higher surgical specialization [22, 45-49].

In the OCHRA paradigm, technical errors are classified as consequential (need remedial action by surgeon) and inconsequential [16, 21, 22]. Any action or omission that causes an adverse event or increases the time of surgical procedure by necessitating a corrective action that falls outside the ‘acceptable limits’ constitutes a consequential error. Inconsequential errors are actions or omissions that increase likelihood of negative consequence and under slightly changed circumstances could result in an adverse effect on patient outcome. Inconsequential errors are important as they serve as ‘near misses’ providing key learning opportunities for reduction of future adverse events [11, 15-20].

Technical errors associated with inability of the surgeon to execute the component steps in the correct order are categorized as ‘procedural error modes,’ while ‘execution error modes’ reflect ineffective/traumatic manipulations [15, 16, 22]. Surgical trainees committed twice the incidence of technical errors than consultant/attending surgeons [16, 22].

Underling mechanisms which provide a deeper understanding of the likelihood of occurrence of technical errors were reported in some studies, e.g., applying excessive force, incorrect order of steps, concentration lapses, misjudgements, poor instrument selection etc., have been identified as factors. Several hazard zones have also been described (Table 1) [15, 16, 21, 22, 25-30] and difficult tasks highlighted [27, 34]. OCHRA enables differentiation between LCs and P-GCs. Learning an operation goes beyond cognitive knowledge, by the individual becoming able to execute the procedure safely, without having to think about it. In this process, the surgeon progresses from the controlled conscious mode (exhausting and cerebrally intensive and subject to fatigue) to the automatic mode, characterized by smooth effortless execution [49].

The study by Miskovic et al. which evaluated the performance of specialists executing live operations in the operating room, confirmed the validity of OCHRA in adjudicating surgical performance at a specialist level and suggested that this method could be implemented for competency assessment within a clinical training program [28]. Potentially, it can also be used for recertification and re-validation.

**Fig. 4:**
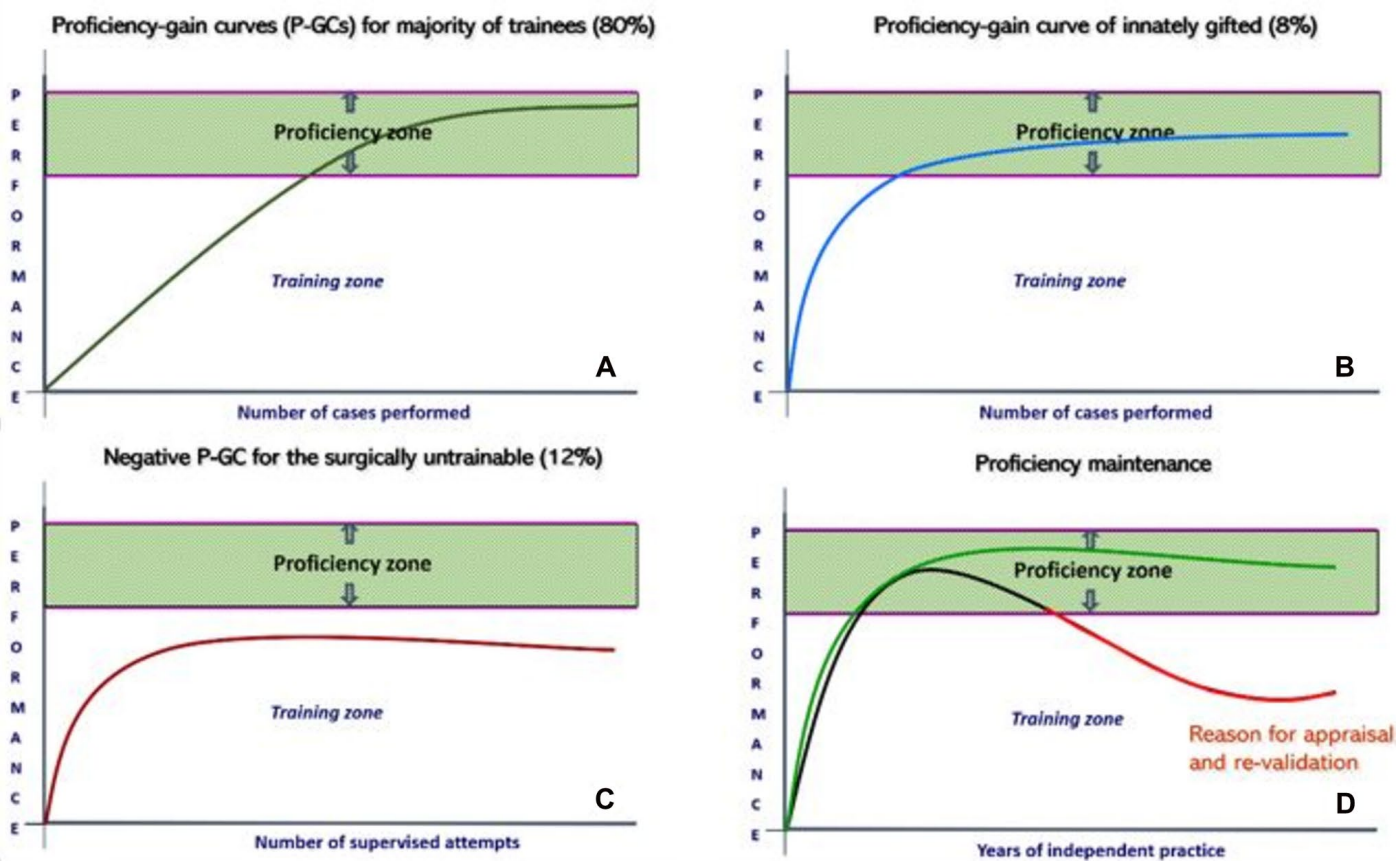
Proficiency gain curves defined by OCHRA: **A** attainment of the proficiency zone by the majority of trainees (80%) for a specific operation; **B** earlier attainment of the proficiency zone by naturally gifted trainees with high level innate aptitude for the same operation as in (**A**); **C** inability to reach the proficiency zone by surgically untrainable surgical trainees (11%), who should be identified at an early stage and advised accordingly; **D** loss of proficiency by previously competent surgeons usually due to disease including alcoholism and other addiction

Equally important, the review highlights the current limitations of OCHRA including its labor-intensive nature involving human factors scientists using established criteria to identify and categorize errors from unedited videos of operations [15, 16, 21-42]. In this respect, the OCHRA will eventually benefit by progress in AI and ML [50]. This development is considered essential for the wider uptake of OCHRA. The review confirms that OCHRA by its documentation and characterisation of errors enacted by operator, constitutes a valid technique for objective assessment of competence in the execution of operations at both consultant and trainee levels (Fig. 4).

## Conclusions

The resulting increased uptake and use of OCHRA would enhance patient outcome after surgery in routine hospital surgical practice and surgical training, aside from being a useful tool for privileging, accreditation and re-validation. The low uptake of OCHRA despite its ability to assess execution quality of operations is attributed to its labor-intensive nature involving human factors (cognitive engineering) expertise. This issue can only be resolved by development of smart video recorders equipped with AI and ML based on incorporated and/or WIFI-accessible huge data sets of unedited recorded operations.
